# Neuropeptide Y and Derivates Are Not Ready for Prime Time in Prostate Cancer Early Detection

**DOI:** 10.1016/j.euros.2024.06.008

**Published:** 2024-06-24

**Authors:** Jonathan Maurer, Philippe J. Eugster, Kiana Collins, Céline Vocat, Jason Oke, Brian Nicholson, Arnas Rakauskas, Eric Grouzmann, Massimo Valerio

**Affiliations:** a Service of Clinical Pharmacology, Lausanne University Hospital and University of Lausanne, Lausanne, Switzerland; b Nuffield Department of Primary Care Health Sciences, Radcliffe Primary Care Building, Radcliffe Observatory Quarter, University of Oxford, Oxford, UK; c Service of Urology, Department of Surgery, Lausanne University Hospital and University of Lausanne, Lausanne, Switzerland; d Service of Urology, Department of Surgery, Geneva University Hospital and University of Geneva, Geneva, Switzerland

**Keywords:** Biomarker, Diagnosis, Neuropeptide Y, Pro-neuropeptide Y, Prostate cancer, Prostate-specific antigen, Quantitative spectrometry

## Abstract

Neuropeptide Y (NPY) and related peptides have been proposed as promising biomarkers for the diagnosis of prostate cancer by previous immunoassays and immunohistochemical studies. In this study, we evaluated the additional value of NPY and related peptides compared with prostate-specific antigen (PSA). We performed a comprehensive analysis of NPY, its precursors, and metabolite concentrations in both plasma and tissue samples from 181 patients using a highly specific liquid chromatography tandem mass spectrometry method. Compared with PSA, NPY and related peptides (NPYs) were less accurate at diagnosing significant prostate cancer. Combinations of NPYs in a stepwise approach did not improve a model that would be beneficial for patients. NPY may be beneficial for patients presenting with a PSA concentration in the gray area between 4 and 9 ng/ml, but the strength of this conclusion is limited. Thus, the use of NPYs as standalone or in combination with other variables, such as PSA, prostate volume, or age, to improve the diagnosis is not supported by our study.

**Patient summary:**

This study evaluated neuropeptide Y (NPY) of the family of endogenous peptides as a new biomarker to diagnose prostate cancer. We found that NPY in a patient’s blood was not more helpful at diagnosing prostate cancer than the standard prostate-specific antigen blood test. Further research is needed to explore the potential of NPY and related peptides in specific subgroups of patients.

In 2022, the European Community recommended to implement a strategy for the early detection of prostate cancer (PCa). Although prostate-specific antigen (PSA) represents the first step in a risk-adapted strategy, it is well known that PSA lacks specificity in PCa diagnosis as this biomarker is organ specific, but not cancer specific. Consequently, there are significant efforts to explore alternative biomarkers that are independent of prostatic normal tissue [1].

Neuropeptide Y (NPY) and its precursor ProNPY are mainly involved in food intake, blood pressure regulation, and energy homeostasis, by stimulating five G protein-coupled receptors (Y1 to Y6) [2]. Effects related to tumor progression have also been highlighted, with NPY and related peptides being involved in cell proliferation, matrix invasion, and angiogenesis [3]. In the context of PCa, these peptides have been proposed as promising biomarkers to improve PCa diagnosis and prognosis [4], [5]. Previous studies suggesting the role of this class of biomarkers relied on genomic, proteomic, and immunohistochemistry analyses. However, these are adapted to evaluate NPY-like expression in prostatic tissue but are not suited to selectively quantify NPY in blood samples. This has hampered the possibility to test these biomarkers in early detection. To address these limitations, we validated a liquid chromatography tandem mass spectrometry assay measuring selectively endogenous plasma NPY and its main precursors and fragments (Supplementary Fig. 1). The innovative sample preparation was adapted from our previously published work [6], [7], [8]. We conducted a single-center study to evaluate whether NPYs help identify significant PCa at an early stage.

Biopsy-naïve patients, 40–80 yr old, undergoing magnetic resonance fusion prostate biopsy for a suspicion of PCa and patients undergoing transurethral resection of the prostate for lower urinary tract symptoms related to benign prostatic hyperplasia were invited to participate in the study. We included these two groups of patients to allow for tissue characterization. Patients’ characteristics are outlined in Supplementary Table 1.

For the purpose of the study, clinically significant disease was defined as Gleason score ≥3 + 4 and/or positive volume threshold defined as maximum cancer core length ≥10 mm on prostate biopsy and/or >5% positive chips on transurethral resection of the prostate (threshold 1 [Th1]). Sensitivity analyses were planned using alternative thresholds of cancer significance to reflect the open debate about this:

1. Threshold 2 (Th2): any Gleason score ≥3 + 4
2. Threshold 3 (Th3): any Gleason score ≥4 + 3

The performance of each variable to discriminate clinically significant from insignificant PCa was evaluated using the areas under the curve (AUCs; Supplementary Table 2) [9].

A total of 124 patients underwent prostate biopsy and 57 underwent transurethral resection of the prostate within the study period from January 4, 2019 to June 18, 2021. Patient's characteristics are in Supplementary Table 1. Overall, 79 (44%) patients were diagnosed with clinically significant PCa. This varied from 72 to 22 according to the threshold used. All NPYs but NPY1-39 were measurable in the plasma and tissue of the 181 patients, with no correlation between them, nor with PSA, age, or prostate volume (Supplementary Fig. 2). Plasma NPY1-36 and NPY3-36 showed AUCs between 0.6 and 0.7 depending on the thresholds used (Fig. 1). Plasma CPON, NPY1-35, NPY3-35, NPY1-37, and ProNPY showed AUCs above 0.6 only when the cohort was restricted to Th3. Considered alone, PSA levels, age, and prostate volume showed higher AUCs than NPYs.

**Fig. 1:**
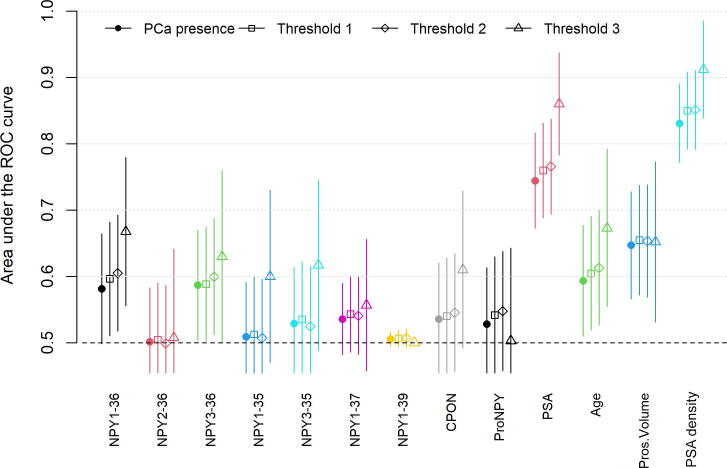
AUCs of the different variables, depending on the threshold considered. Threshold 1: Gleason score ≥3 + 4 and/or positive volume threshold defined as maximum cancer core length ≥10 mm on prostate biopsy and/or >5% positive chips on transurethral resection of the prostate; threshold 2: any Gleason score ≥3 + 4; threshold 3: any Gleason score ≥4 + 3. AUC = area under the curve; NPY = neuropeptide Y; PCa = prostate cancer; Pros.Volume = prostate volume; PSA = prostate-specific antigen; ROC = receiver operating characteristic.

A stepwise procedure was used to create a model based on multiple variables, to evaluate whether some of the NPYs might improve the discrimination of clinically significant PCa. This model was compared with a reference model based on PSA, age, and prostate volume.

The retained predictive variables were prostate volume, age, PSA, NPY2-36, NPY3-36, NPY1-37, and ProNPY. Increased age, PSA, NPY3-36, and NPY1-37, and decreased NPY2-36 and ProNPY were associated with an increased risk of clinically significant PCa. We found no difference between the AUCs of the NPYs and the reference models (86.8 and 85.5, respectively, *p* = 0.26). Thus, our data do not support the use of NPYs in combination with PSA, age, and prostate volume to improve the diagnosis of clinically significant PCa.

In a subanalysis, we explored whether NPYs provided a diagnostic value for patients with specific levels of PSA. When looking at patients with PSA values between 4 and 9 ng/ml (*n* = 66), we found that these PSA levels were negatively associated with clinically significant PCa. The variables retained by the stepwise selection are expressed in the following equation.

$$\mathrm{Pr}\left(Y\;=\;1\right)=\;1\;+{\exp\left(-Xb\right)}^{-1}$$

Here, Xb=9.59 – 2.87 × log(prostate volume) + 0.12 × (age [yr]) – 2.02 × log(PSA) – 0.83 × log(NPY2-36) + 1.60 × log(NPY1-37).

Although we observed some evidence of improved AUC rates (Fig. 2), differences between the NPY and the reference models did not meet conventional levels of statistical significance (85.7 and 78.2, respectively, *p* = 0.12). The models were also compared at fixed sensitivities (Supplementary Table 3).

**Fig. 2:**
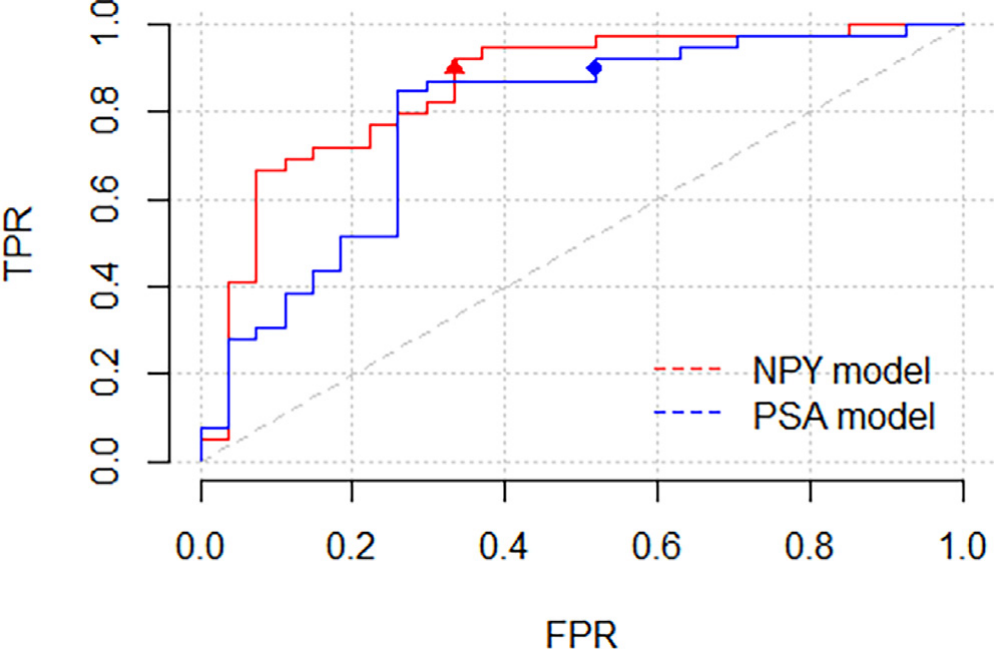
AUCs of the two models when applied on patients of threshold 1 with PSA level between 4 and 9 ng/ml. AUC = area under the curve; FPR = false positive rate; PSA = prostate-specific antigen; TPR = true positive rate.

Unfortunately, the small cohort of patients with PSA values between 4 and 9 ng/ml implies a high risk of data overfitting and does not allow for the creation of a robust statistical model. Thus, the quantification of NPY2-36 and NPY1-37 may help further categorize patients with moderate PSA values between 4 and 9 ng/ml. However, to justify creating a clinical decision rule and evaluate data overfitting, further confirmation in a larger cohort is needed to gain a better understanding of NPYs on a continuous range of PSA values.

In tissue, ProNPY was the sole NPY that exhibited a significant correlation between tissue and plasma concentrations (Supplementary Fig. 4 and 5). This supports either tumoral secretion of proNPY important enough to modify the plasma concentration or lower stability of the other NPYs because of circulating proteases. Thus, even in patients with highly aggressive PCa (Th3), NPYs measured in plasma mainly originated from secretion by the sympathetic nerves and the adrenal medulla.

Within this well-selected prospective cohort, our study confirms previous findings that NPYs are expressed in PCa but highlights the limitations of an NPY assay for early detection of PCa. The discrepancies observed between our findings and the previous studies are likely a consequence of the innovative use of a highly specific and dedicated mass spectrometry method. However, for patients showing levels of PSA between 4 and 9 ng/ml, the combination of NPY2-36 and NPY1-37 with PSA density and age in a statistical model showed promising diagnostic improvement. Before considering these findings usable in a clinical context, the results should be confirmed in a larger cohort of patients to avoid data overfitting and better understand the diagnostic performance of NPY across a range of PSA values. In the meantime, NPYs should not be used as a diagnostic tool for PCa.

  ***Author contributions*:** Jonathan Maurer had full access to all the data in the study and takes responsibility for the integrity of the data and the accuracy of the data analysis.

  *Study concept and design*: Valerio, Grouzmann, Eugster.

*Acquisition of data*: Maurer, Valerio, Vocat, Rakauskas.

*Analysis and interpretation of data*: Maurer, Valerio, Grouzmann, Eugster.

*Drafting of the manuscript*: Maurer, Grouzmann, Eugster, Valerio, Collins.

*Critical revision of the manuscript for important intellectual content*: Maurer, Grouzmann, Eugster, Valerio, Collins, Oke, Nicholson, Vocat, Rakauskas.

*Statistical analysis*: Collins, Oke, Nicholson, Maurer.

*Obtaining funding*: Valerio, Grouzmann, Eugster, Maurer.

*Administrative, technical, or material support*: None.

*Supervision*: Valerio, Grouzmann, Eugster, Oke, Nicholson.

*Other*: None.

  ***Financial disclosures:*** Jonathan Maurer certifies that all conflicts of interest, including specific financial interests and relationships and affiliations relevant to the subject matter or materials discussed in the manuscript (eg, employment/affiliation, grants or funding, consultancies, honoraria, stock ownership or options, expert testimony, royalties, or patents filed, received, or pending), are the following: None.

  ***Funding/Support and role of the sponsor*:** This project was supported by the Swiss Cancer League (grant number KLS-4283-08-2017 to Massimo Valerio, Philippe J. Eugster, and Eric Grouzmann). The Bryn Turner-Samuels Foundation (Lausanne, Switzerland) is also gratefully acknowledged for the funding of Jonathan Maurer’s doctoral thesis.

  ***Acknowledgments*:** The authors warmly thank Marielle Dunand for her technical help, and Shannon Mechoullam and Jean-Paul Rivals for the database management.
