# Supplementary material for: Neuropeptide Y and Derivates Are Not Ready for Prime Time in Prostate Cancer Early Detection
Source: Eur Urol Open Sci. 2024 Jun 24;66:12–5. doi: 10.1016/j.euros.2024.06.008 (PMC11254586; doi:10.1016/j.euros.2024.06.008)
Supplement: Supplementary Data 1 [file mmc1.docx]

**NPY and derivates are not ready for prime time in prostate cancer early detection: supplementary material**

# Material and methods

## *Chemical and reagents*

Prionex protein stabilizer solution was purchased from Sigma-Aldrich (Buchs, Switzerland). FastPrep-24 5G with 2 ml RNAse-DNAse–free lysing matrix A tubes were ordered from Mpbio (Santa Ana, California, USA). Pierce BCA Protein Assay Kit was purchased from Thermo Fisher Scientific (Waltham, Massachusetts, USA). Other chemicals and reagents were used as described in previous studies [1,2].

## *Patients and sample collection*

The recruitment of 206 men included in this study was approved by the ethical committee of the Etat de Vaud (CER-VD study 2017-00366). Of the 206 men, 25 had incomplete data and were not kept in the study, leading to a total cohort of 181 patients.

The following data were collected: age, date of examinations, prostate-specific antigen (PSA) level, neuropeptide Y (NPY) tissue and plasma concentration, clinical examination findings (digital rectal examination), imaging findings (transrectal and abdominal ultrasound, prostate magnetic resonance, computed tomography (CT) scan, bone scintigraphy, and positron emission tomography CT when available), and disease stage and characteristics (including Gleason score, maximum cancer core length, total cancer core length, etc.). The presence of the tumor was assessed through standard histological verification by two expert board-certified urogenital pathologists, according to the International Society of Urological Pathology recommendations. All the data were stored on a password-protected computer database prior to the measure of NPY and related peptides (NPYs), to ensure a blinded experiment.

**Supplementary Table 1 – Clinical characteristics of the 181 patients included in the final cohort**

| Variable | Biopsy | TURP | Overall |
| --- | --- | --- | --- |
| Patients, *n* | 124 | 57 | 181 |
| Age at diagnosis (yr), median (interquartile range) | 66 (60–72) | 68 (64–72) | 67 (61–72) |
| Prostate-specific antigen at diagnosis (ng/ml), median (interquartile range) | 8 (5–11) | 3 (1–6) | 6 (4–10) |
| Prostate volume (ml), median (interquartile range) | 42 (34–52) | 53 (35–76) | 45 (34–60) |
| Prostate-specific antigen density (ng/ml^2^), median (interquartile range) | 0.18 (0.11–0.32) | 0.06 (0.03–0.09) | 0.12 (0.06–0.26) |
| Maximum PI-RADS score for the index lesion, *n* (%) |  |  |  |
| 1 | 2 (2) | 1 (2) | 3 (2) |
| 2 | 11 (9) | 11 (19) | 22 (12) |
| 3 | 5 (4) | 2 (4) | 7 (4) |
| 4 | 58 (47) | 1 (2) | 59 (33) |
| 5 | 47 (38) | 1 (2) | 48 (27) |
| NA | 1 (1) | 41 (72) | 42 (23) |
| Gleason score, *n* (%) |  |  |  |
| Benign | 36 (29) | 54 (95) | 90 (50) |
| 3 + 3 | 17 (14) | 2 (4) | 19 (10) |
| 3 + 4 | 49 (40) | 1 (2) | 50 (28) |
| 4 + 3 | 8 (6) | 0 | 8 (4) |
| 4 + 4 | 5 (4) | 0 | 5 (3) |
| 4 + 5 | 9 (7) | 0 | 9 (5) |
| Maximum diameter of the index lesion (mm), median (interquartile range) | 28 (14–46) | - | 28 (14–46) |
| Number of biopsy cores, median (interquartile range) | 15 (12–20) | 6 (9–11) | 13 (10–17) |
| Prostate cancer aggressiveness, *n* |  |  |  |
| PCa absent | 36 | 54 | 90 (50) |
| PCa Present | 88 | 3 | 91 (50) |
| Threshold 1 | 78 | 1 | 79 (44) |
| Threshold 2 | 71 | 1 | 72 (40) |
| Threshold 3 | 22 | 0 | 22 (12) |

NA = not available; PCa = prostate cancer; PI-RADS = Prostate Imaging Reporting and Data System; TURP = transurethral resection of the prostate.

## *Sample extraction and analysis*

The sample preparation for the tissue samples was adapted from the validated method by Eugster et al [1] and Vocat et al [2], and included a solid phase extraction (SPE) followed by immunoenrichment of the peptides, and a second SPE. The handling of tissue samples followed the strategies presented in the study of Maurer et al [3].

Tumor and transurethral resection of the prostate (TURP) samples were cut into small pieces on dry ice and placed into lysing matrix A tube. Ice cold 300 µl lysis buffer (6M guanidinium chloride, nonyl-β-d-glucopyranoside [NG] 0.1%, formic acid [FA] 0.1%,prionex 0.01%) was added into the tubes. Tubes were set in refrigerated TissueLyser LT (Qiagen, Hilden, Germany) that was run at an oscillation frequency of 50 Hz for 3 min. The tubes were then centrifuged at 21 250 × *g* for 10 min at 4°C. The whole content of the lysing matrix tubes was transferred into 8-ml Minisorp tubes and kept on ice. Lysing matrix tubes were rinsed three times with 233 µl ice cold lysis buffer and pooled in the 8-ml Minisorp tubes. Samples were sonicated using a Branson Ultrasonics Sonifier SFX250 Cell Disruptorsat at the maximum amplitude (70%) for 1 min on ice. After centrifugation, supernatants were transferred in 2-ml polypropylene Eppendorf. A second sonication method was performed using 1 ml lysis buffer. Supernatants were pooled and stored at –80°C until extraction.

Five microliters of sonicated tumor samples and 100 µl of sonicated TURP samples were diluted with 985 and 890 µl dilution buffer (acetic acid [AcOH] 2 M, NG 0.1%,prionex 0.01%), respectively, and completed with 10 µl internal standard. Waters (Milford, Massachusetts, USA) Oasis HLB 96-well plate, 30 µm (5 mg), designed for SPE, was conditioned with 500 µl acetonitrile (ACN) and equilibrated with 500 µl AcOH 2 M. After loading the samples, SPE column was washed with 500 µl AcOH 2M, 500 µl ammonium hydroxide 1%, and 500 µl water. Analytes were eluted with twice 100 µl ACN 45%, NG 0.1%, and FA 0.1%, and were dried.

Tissue samples were reconstituted in 10 µl ACN 20%, NG 0.1%, and FA 1%, and completed with 500 µl 0.1% NG charcoal stripped plasma. Both plasma and extracted tissue samples solubilized in plasma were then extracted and analyzed using UHPLC-MS/MS, as described in the studies of Eugster et al [1] and Vocat et al [2].

To compensate for the variations of the tissue mass, the NPY concentrations found in the tissue were normalized by the protein content measured by the BCA Protein Assay Kit. For both tumor and TURP samples, 10 µl of sonicated tissue were quantified following the instruction of the kit.

## *Statistical analyses*

R version 4.3.0 (R Foundation for Statistical Computing, Vienna, Austria), the pRoC package [4], and Prism 9.1.0 for Windows (GraphPad, San Diego, CA, USA) were used to produce all the statistical analyses.

The populations used to develop the models were the prostate cancer (PCa)-negative population (*n* = 90) with no cancer detected, and the clinically significant PCa population (threshold 1, *n* = 79) with patients showing clinically significant cancer confirmed by biopsy. The outcome was the presence of PCa.

The plasma concentrations under the lower limit of quantification (LLOQ) were set at half the LLOQ of the corresponding analyte [5]. NPY1-39 had only one value above the LLOQ and thus was not considered a candidate predictor in the model. The variables were right skewed and normalized using natural logarithm for statistical evaluation.

The area under the receiver operating characteristic curve (AUC) was obtained for every variable to estimate their ability to discriminate between cohorts. Collinearity was checked using the variance inflation factor [6].

A stepwise selection procedure based on the Akaike information criterion was used to choose the best fitting, parsimonious model for PCa. The logistic regression model package in R (lrm()) was used to get the bootstrap estimates of model optimism.

# Results

## *NPY metabolism*


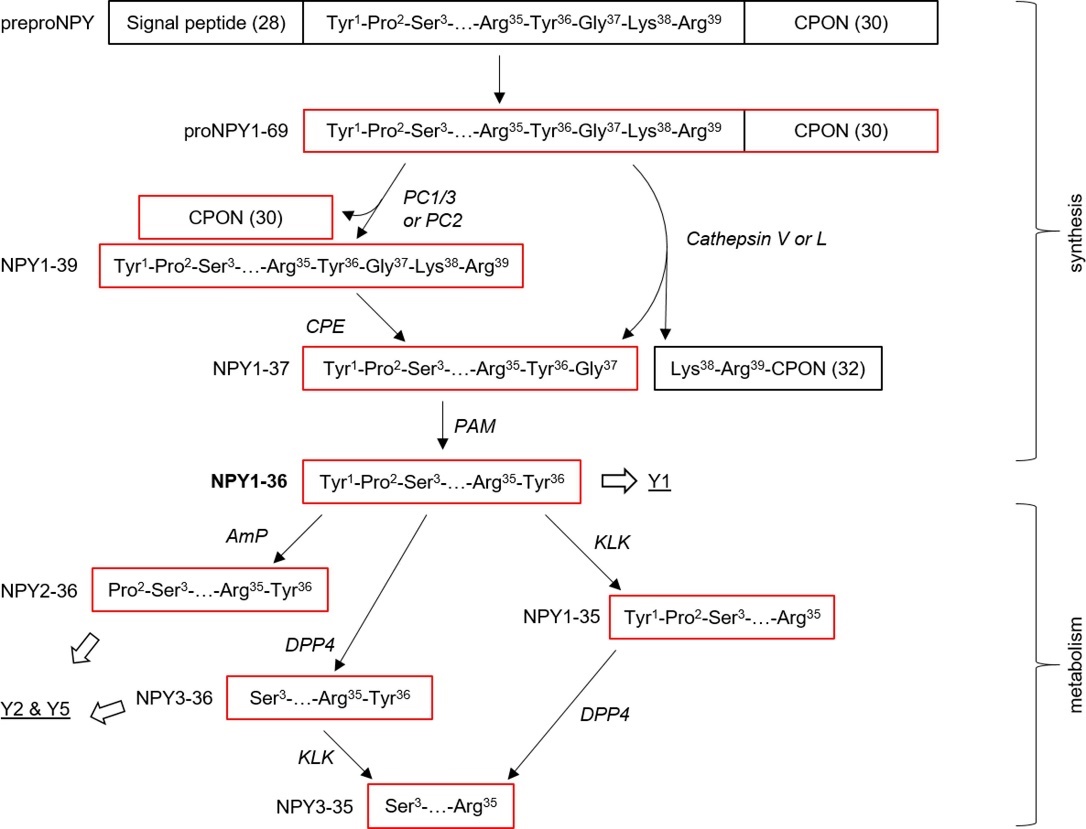


**Supplementary Fig. 1 – Metabolism of the NPY, its precursors and fragments, as described in the study of Eugster et al [1].**

**NPY = neuropeptide Y.**

## *Plasma PSA and NPYs concentrations distribution*

**Supplementary Fig. 2 – NPY concentrations found in the plasma of the 181 patients in the cohort. Whiskers are set at 2.5% and 97.5%.**

**NPY = neuropeptide Y; PCa = prostate cancer.**

## *Correlation between the variables*


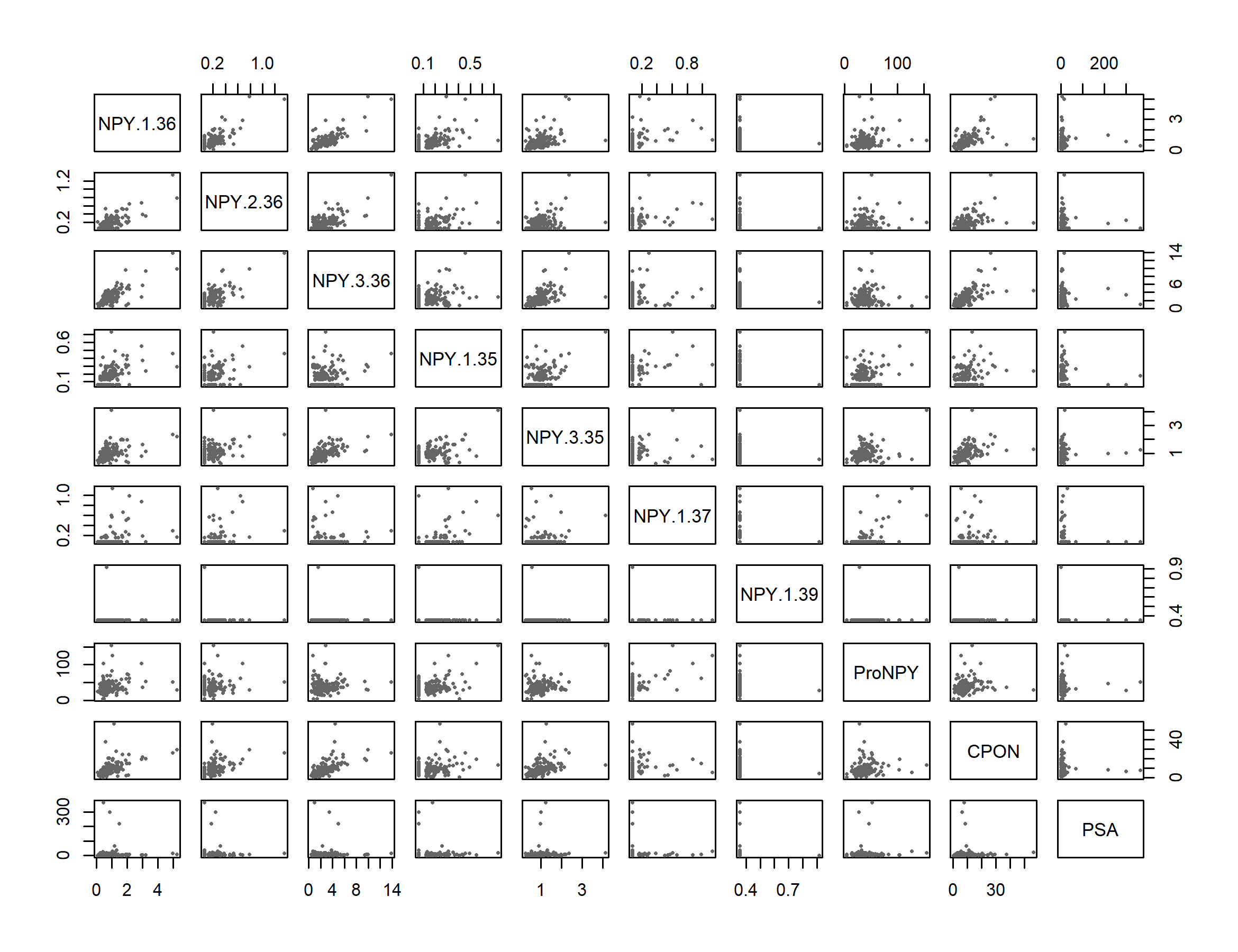


**Supplementary Fig. 3 – Correlation matrix of the NPYs and PSA.**

**NPY = neuropeptide Y; PSA = prostate-specific antigen.**

## *Areas under the receiver operating characteristic curves*

**Supplementary Table 2 – AUCs of variables measured in patients to detect significant cancer, depending on the aggressiveness**

| Variable | PCa presence (*n* = 91) | Threshold 1 (*n* = 79) | Threshold 2 (*n* = 72) | Threshold 3 (*n* = 22) |
| --- | --- | --- | --- | --- |
|  | AUC (95% CI) | AUC (95% CI) | AUC (95% CI) | AUC (95% CI) |
| log(NPY1-36) | 58.1 (49.8–66.5) | 59.6 (51.1–68.2) | 60.5 (51.8–69.3) | 66.8 (55.6–78.0) |
| log(NPY2-36) | 50.1 (42.0–58.3) | 50.5 (42.0–59.0) | 49.9 (41.2–58.7) | 50.8 (37.5–64.1) |
| log(NPY3-36) | 58.7 (50.4–67.0) | 58.8 (50.2–67.4) | 60.0 (51.2–68.7) | 63.0 (50.0–76.0) |
| log(NPY1-35) | 50.9 (42.6–59.1) | 51.3 (42.7–59.9) | 50.7 (41.9–59.6) | 60.0 (47.0–73.0) |
| log(NPY3-35) | 52.9 (44.5–61.4) | 53.5 (44.7–62.3) | 52.4 (43.4–61.5) | 61.7 (48.8–74.6) |
| log(NPY1-37) | 53.6 (48.2–58.9) | 54.3 (48.6–60.0) | 54.1 (48.2–59.9) | 55.7 (45.8–65.6) |
| log(NPY1-39) | 50.5 (49.5–51.6) | 50.6 (49.4–51.9) | 50.7 (49.3–52.1) | 50.0 (50.0–50.0) |
| log(CPON) | 53.5 (45.1–62.0) | 54.0 (45.3–62.8) | 54.5 (45.7–63.4) | 61.0 (49.2–72.8) |
| log(ProNPY) | 52.8 (44.3–61.3) | 54.2 (45.4–63.0) | 54.8 (45.8–63.8) | 50.3 (36.4–64.3) |
| log(Prost. volume) | 64.7 (56.7–72.8) | 65.5 (57.2–73.7) | 65.4 (56.9–73.8) | 65.2 (53.1–77.3) |
| log(PSA) | 74.4 (67.3–81.6) | 76.0 (68.9–83.1) | 76.6 (69.4–83.7) | 86.0 (78.3–93.7) |
| Age | 59.1 (50.7–67.4) | 60.1 (51.6–68.7) | 61.6 (52.9–70.3) | 68.5 (56.8–80.3) |

AUC = area under the receiver operating characteristic curve; CI = confidence interval; NPY = neuropeptide Y; PCa = prostate cancer; PSA = prostate-specific antigen.

## *Combining NPYs and PSA to increase diagnosis performance*

Equation of the model based on the population showing clinically significant PCa, with the variables retained by the stepwise selection, is the following:

$$\Pr\left( Y = 1 \right)= 1 +{\exp\left( -Xb \right)}^{-1}$$

$Here,Xb=$3.56 – 2.13 × log(prostate volume) + 0.07 × (age) + 1.53 × log(PSA) – 0.82 × log(NPY2-36) + 0.87 × log(NPY3-36) + 0.89 × log(NPY1-37) – 0.88 × log(ProNPY).

The reference model was created using only PSA, age, and prostate volume, on the same population where:

Xb = 1.03 – 2.14 × log(prostate volume) + 0.07 × (age at inclusion [in years]) + 1.51 × log(PSA)

## *Model based on patients with PSA between 4 and 9 ng/ml*

**Supplementary Table 3 – AUCs obtained for the NPY and PSA models on patients with clinically significant prostate cancer (threshold 1) and PSA level between 4 and 9 ng/ml**

| Sensitivity | AUCs | Specificity | PPV | NPV |
| --- | --- | --- | --- | --- |
| 0.90 | 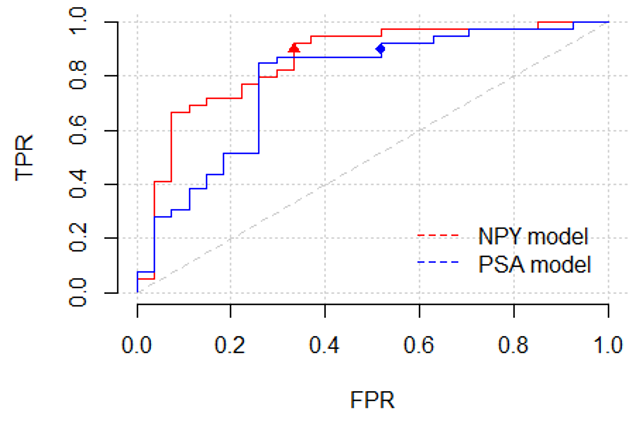 | NPY: 0.67  PSA: 0.48 | NPY: 79.8%  PSA: 71.4% | NPY: 82.3%  PSA: 76.9% |
| 0.95 | 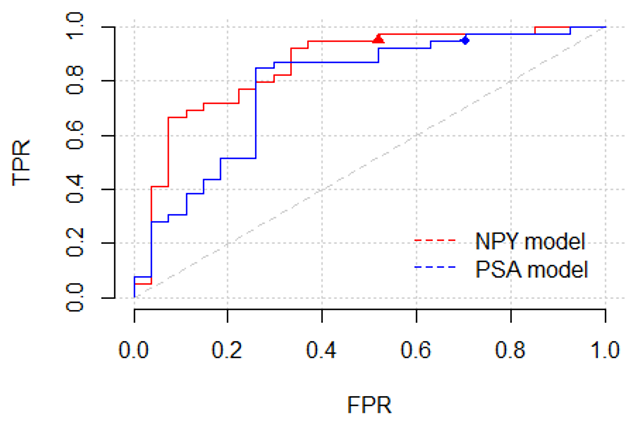 | NPY: 0.48  PSA: 0.30 | NPY: 72.5%  PSA: 66.2% | NPY: 86.9%  PSA: 80.6% |

AUC = area under the receiver operating characteristic curve; FPR = false positive rate; NPV = negative predictive value; NPY = neuropeptide Y; PPV = NPV = positive predictive value; PSA = prostate-specific antigen, TPR = true positive rate.

The NPY model showed an AUC of 0.86 before and 0.81 after the correction for the optimism.

## *NPYs in tissue*

NPYs were quantified in tissues, and their concentrations were normalized using the total protein content.

**Supplementary Fig. 4 – NPY concentration in the tissue, depending on the threshold. Whiskers are set at 2.5% and 97.5%.**

**NPY = neuropeptide Y; PCa = prostate cancer.**

The correlations between the concentration of a peptide in the tissue and the plasma are expressed in Supplementary Figure 5.

**Supplementary Fig. 5 – Correlations between tissue and plasma concentrations of the NPYs, calculated in the population showing clinically significant PCa.**

**NPY = neuropeptide Y; PCa = prostate cancer.**

# References

1. Eugster PJ, Maurer J, Vocat C, et al. Proneuropeptide Y and neuropeptide Y metabolites in healthy volunteers and patients with a pheochromocytoma or paraganglioma. Clin Chim Acta 2022;534:146–55.
2. Vocat C, Dunand M, Hubers SA, et al. Quantification of neuropeptide Y and four of its metabolites in human plasma by micro-UHPLC-MS/MS. Anal Chem 2020;92:859–66.
3. Maurer J, Grouzmann E, Eugster PJ. Tutorial review for peptide assays: An ounce of pre-analytics is worth a pound of cure. J Chromatogr B Analyt Technol Biomed Life Sci 2023;1229:123904.
4. Robin X, Turck N, Hainard A, et al. pROC: an open-source package for R and S+ to analyze and compare ROC curves. BMC Bioinformatics 2011;12:77.
5. Johnson JR. Methods for handling concentration values below the limit of quantification in PK studies. PhUSE US Connect. 2018, 1-9.
6. Kleinbaum DG, Kupper LL, Muller KE, Nizam A. Applied regression analysis and other multivariable methods. ed. 3. Belmont, CA: Duxbury Press; 1998.
